# Cost-effectiveness analysis of first-line tislelizumab plus chemotherapy for recurrent or metastatic nasopharyngeal cancer

**DOI:** 10.3389/fphar.2023.1265784

**Published:** 2023-10-30

**Authors:** Zhengda Pei, Ningping Xiao, Pei Yang

**Affiliations:** ^1^ Hunan Cancer Hospital, The Affiliated Cancer Hospital of Xiangya School of Medicine, Central South University, Changsha, Hunan, China; ^2^ Graduate Collaborative Training Base of Hunan Cancer Hospital, Hengyang Medical School, University of South China, Hengyang, Hunan, China; ^3^ Key Laboratory of Translational Radiation Oncology of Hunan Province, Changsha, Hunan, China

**Keywords:** recurrent or metastatic nasopharyngeal carcinoma, tislelizumab, cost-effectiveness, quality-adjusted life-years, incremental cost-effectiveness ratio

## Abstract

**Introduction:** The RATIONALE-309 trial confirmed the significant efficacy and safety of tislelizumab plus chemotherapy in patients with recurrent or metastatic nasopharyngeal carcinoma (R/M NPC). However, the economic benefits of this regimen are unclear. Therefore, this study aimed to evaluate the cost-effectiveness of adding tislelizumab to chemotherapy for R/M NPC from the perspective of the Chinese healthcare system.

**Methods:** A Markov model was established to simulate the costs and outcomes of tislelizumab plus chemotherapy versus chemotherapy. The survival data came from the RATIONALE-309 trial. Only direct medical costs were considered, and utility values were referred to the literature. The incremental cost-effectiveness ratio (ICER) was used as the main outcome measure. Sensitivity analysis was performed to assess the effect of parameter uncertainty on the model. Additionally, subgroup analyses were performed.

**Results:** The basic analysis showed that the cost of tislelizumab plus chemotherapy ($33,693) was $17,711 higher than that of chemotherapy ($15,982), but it also gained 1.05 QALYs more (2.72 QALYs vs. 1.67 QALYs), with an ICER of $16,859/QALY, which was lower than the willing-to-pay (WTP) of $36,289/QALY. The factors that most influenced the model were the utility of PD, the cost of tislelizumab, and the risk of platelet count decreased in tislelizumab plus chemotherapy group. The subgroup analysis also demonstrated that tislelizumab plus chemotherapy was cost-effective in the whole population regardless of EBV DNA level and PD-L1 expression level.

**Conclusion:** Compared with chemotherapy alone, tislelizumab plus chemotherapy was cost-effective for the treatment of R/M NPC in China.

## 1 Introduction

Nasopharyngeal carcinoma (NPC) is a form of epithelial cancer derived from the inner nasopharyngeal mucosa that is etiologically distinct from other forms of head and neck cancer such that different treatment strategies are warranted for affected patients (Chen et al., 2019). There were an estimated 133,000 NPC diagnoses and 80,000 deaths in 2020 alone, the majority of which were concentrated in South China, Southeast Asia, and North Africa (Sung et al., 2021). While excellent local control has been achieved in NPC patients via intensity-modulated radiation therapy (IMRT), approximately 10% of newly diagnosed patients present with synchronous metastases. Moreover, 10%–20% of patients ultimately develop local or nodal recurrence following initial treatment, while 15%–30% develop distant metastatic disease (Lee et al., 2019; Wong et al., 2021). Platinum-based chemotherapeutic regimens have been shown to improve the progression-free and overall survival (PFS and OS) of NPC patients, with median PFS and OS durations of 5–7 months and 20–29 months, respectively, although the outcomes associated with first-line chemotherapy alone remain unsatisfactory (Wang and Tan, 1991; Jin et al., 2012; Zhang et al., 2016). Recurrent or metastatic NPC (R/M NPC) thus remains a major therapeutic challenge.

Epstein-Barr virus (EBV)-related NPC is an “immune-hot” tumor that is often accompanied by high levels of PD-L1 expression and infiltration by large quantities of non-malignant lymphocytes (Wong et al., 2021). For this reason, immune checkpoint inhibitor (ICI) treatment has shown excellent efficacy when used to treat R/M NPC patients. Camrelizumab is a monoclonal humanized antibody reported to significantly prolong the PFS and OS of R/M NPC patients. In the multicenter randomized phase III CAPTAIN-1st trial (NCT03707509), camrelizumab plus chemotherapy was associated with the prolongation of patient PFS by 2.9 months relative to placebo plus GP (9.7 vs. 6.9 months; hazard ratio (HR), 0.54; 95% confidence interval (CI), 0.39 to 0.76; *p* = 0.0002) (Yang et al., 2021). The median OS in the placebo plus chemotherapy group was 22.6 months, whereas this endpoint was not reached in the camrelizumab group for that trial (HR, 0.67; 95% CI, 0.41–1.11) (Yang et al., 2021). Toripalimab is a humanized monoclonal anti-PD-1 antibody that has also exhibited pronounced efficacy and tolerable safety when used to treat R/M NPC. In the randomized multicenter phase III JUPITER-02 trial (NCT03581786), toripalimab plus chemotherapy was associated with the significant prolongation of patient median PFS relative to placebo plus GP (11.7 vs. 8.0 months; HR, 0.52; 95% CI, 0.36 to 0.74), and the former combination was also associated with a 40% decrease in the risk of death (HR, 0.603; 95% CI, 0.364 to 0.997) (Mai et al., 2021). Camrelizumab and toripalimab have thus both received approval from the National Medical Products Administration (NMPA) of China as treatments for R/M NPC such that they received recommendations as first-line treatments under Chinese Society of Clinical Oncology (CSCO) guidelines (Csco, 2023; Nmpa, 2023).

The results of the RATIONALE-309 (NCT03924986) trial were published in June of 2023. Tislelizumab is a high-affinity humanized monoclonal anti-PD-1 antibody of the IgG4 subtype. In R/M NPC, the combination of tislelizumab plus chemotherapy resulted in the significant prolongation of median PFS relative to placebo plus chemotherapy (9.2 vs. 7.4 months; HR, 0.52; 95% CI; 0.38 to 0.73; *p* < 0.0001) (Yang et al., 2023). The median OS in the placebo plus chemotherapy group was 23.0 months, while this endpoint was not reached in the tislelizumab plus chemotherapy group. These data suggest that tislelizumab represents a promising approach to the treatment of R/M NPC patients.

Despite the satisfactory efficacy of tislelizumab in available trial data, the high costs of immunotherapeutic regimens have led to hesitance among patients and clinicians regarding their application. There is thus a pressing need to adequately balance the costs and efficacy of these innovative therapies. As such, the present analysis was developed to assess the cost-effectiveness of tislelizumab plus chemotherapy as a first-line treatment option for R/M NPC from a Chinese payer perspective.

## 2 Materials and methods

This economic analysis was conducted based on data derived from the RATIONALE-309 trial. This study did not enroll any actual human participants, and no institutional review board approval was required. This study was reported in accordance with CHEERS (Consolidated Health Economic Evaluation Reporting Standards) 2022 (Supplementary Table S1) (Husereau et al., 2022).

### 2.1 Patients and intervention

Patients enrolled in this analysis were randomized at a 1:1 ratio to undergo treatment with tislelizumab (200 mg) plus chemotherapy or placebo plus chemotherapy. Chemotherapy regimens for all patients were comprised of gemcitabine (1 g/m^2^, days 1 and 8) and cisplatin (80 mg/m^2^, day 1) (Supplementary Table S2). Patients received 4-6 cycles of chemotherapy based on their condition. For patients unable to tolerate the administered chemotherapeutic regimen, two dose reductions for each agent were permitted prior to discontinuation. Tislelizumab dose reductions were not permitted. Patients were allowed to temporarily discontinue treatment to recover from adverse events (AEs), but were required to resume treatment within 12 weeks after the most recent dose. Treatment was performed until patients experienced progression, unacceptable toxicity, or permanent discontinuation. All drug administration in the present study was based on the assumption that all patients were 65 years old, 164 cm tall, 65 kg in weight, and had a 1.72 m^2^ body surface area (Zhu et al., 2022). Based on the limitations of the data included in the RATIONALE-309 trial, subsequent treatment strategies were selected based on the 2022 recommendations from the CSCO (Csco, 2023).

### 2.2 Model structure

TreeAge Pro 2022 (TreeAge Software, MA, USA; https://www.treeage.com) was used to construct a Markov model and to conduct the cost-effectiveness analysis for tislelizumab and chemotherapy in R/M NPC discussed herein (Supplementary Figure S1). In the established three-state Markov model, all patients began in the progression-free survival (PFS) state and had a chance to enter the progressive disease (PD) or death states. Each patient was only in one of these states at a time and could not progress to the prior state after entering the next state. Each cycle of this model was 6 weeks in length, and the model had a 10-year time horizon. A 5% annual discounting rate was used for all costs and outcomes in this model (Zhao et al., 2022). Total costs, life-years (LYs), quality-adjusted life-years (QALYs), and incremental cost-effectiveness ratio (ICER) values were used to evaluate treatment cost-effectiveness relative to a willingness-to-pay (WTP) threshold of $36,289/QALY (3 times the *per capita* GDP of China in 2022) (Liu et al., 2022).

### 2.3 Model survival and transition probabilities

PFS and OS data from the RATIONALE-309 trial were extracted using GetData Graph Digitizer (version 2.26; http://www.getdata-graphdigitizer.com/index.php) and utilized to construct a survival model incorporating transition probabilities. The fitting of these data to Weibull, exponential, log-logistic, Gompertz, and log-normal distributions was assessed, with the Akaike information criterion (AIC) and Bayesian information criterion (BIC) ultimately revealing that all curves best fit the Weibull distribution (Supplementary Figure S2 and Supplementary Table S3). R (version 4.1.1, http://www.rproject.org) was then used to approximate the shape (γ) and scale (λ) parameters, and Kaplan-Meier curves were employed as detailed previously by Hoyle and Henley (2011) (Table 1).

**TABLE 1:** Key parameters.

Variable	Mean value (Range)	Reference	Distribution
Clinical parameters			
Weibull survival model for tislelizumab plus chemotherapy			
OS	Scale = 0.005008, Shape = 1.297713	—	—
PFS	Scale = 0.075560, Shape = 0.950960	—	—
Weibull survival model for chemotherapy			
OS	Scale = 0.001060, Shape = 2.027554	—	—
PFS	Scale = 0.034444, Shape = 1.615226	—	—
Rate of post-discontinuation therapy			
Tislelizumab plus chemotherapy group	0.520 (0.416–0.624)	Yang et al. (2023)	Beta
Chemotherapy group	0.720 (0.576–0.864)	Yang et al. (2023)	Beta
Risk for main AEs in tislelizumab plus chemotherapy group			
Lymphocyte count decreased	0.107 (0.086–0.128)	Yang et al. (2023)	Beta
Platelet count decreased	0.206 (0.165–0.247)	Yang et al. (2023)	Beta
Leukopenia	0.206 (0.165–0.247)	Yang et al. (2023)	Beta
Neutropenia	0.214 (0.171–0.257)	Yang et al. (2023)	Beta
Neutrophil count decreased	0.275 (0.220–0.330)	Yang et al. (2023)	Beta
Anemia	0.298 (0.238–0.358)	Yang et al. (2023)	Beta
White blood cell count decreased	0.313 (0.250–0.376)	Yang et al. (2023)	Beta
Risk for main AEs in chemotherapy group			
Lymphocyte count decreased	0.121 (0.097–0.145)	Yang et al. (2023)	Beta
Leukopenia	0.159 (0.127–0.191)	Yang et al. (2023)	Beta
Neutropenia	0.189 (0.151–0.227)	Yang et al. (2023)	Beta
Platelet count decreased	0.258 (0.206–0.310)	Yang et al. (2023)	Beta
Anemia	0.273 (0.218–0.328)	Yang et al. (2023)	Beta
Neutrophil count decreased	0.348 (0.278–0.418)	Yang et al. (2023)	Beta
White blood cell count decreased	0.371 (0.297–0.445)	Yang et al. (2023)	Beta
Utility and disutility			
Utility of PFS	0.650 (0.520–0.780)	Zhu et al. (2022)	Beta
Utility of PD	0.520 (0.416–0.624)	Zhu et al. (2022)	Beta
Disutility of AEs in TC group	0.0070 (0.0056–0.0084)	Zhu et al. (2022)	Beta
Disutility of AEs in chemtherapy group	0.0069 (0.0055–0.0083)	Zhu et al. (2022)	Beta
Cost, $/per cycle			
Treatment cost			
Tislelizumab	778 (622–934)	Real World	Gamma
Gemcitabine	39 (31–47)	Real World	Gamma
Cisplatin	31 (25–37)	Real World	Gamma
Second-line Therapy	77 (62–92)	Real World	Gamma
Cost of AEs			
Leucopenia	100 (80–120)	Tian et al. (2022), Zhu et al. (2022)	Gamma
Lymphocyte count decreased	100 (80–120)	Tian et al. (2022), Zhu et al. (2022)	Gamma
Neutropenia	466 (373–559)	Tian et al. (2022), Zhu et al. (2022)	Gamma
White blood cell count decreased	466 (373–559)	Tian et al. (2022), Zhu et al. (2022)	Gamma
Neutrophil count decreased	466 (373–559)	Tian et al. (2022), Zhu et al. (2022)	Gamma
Anaemia	537 (430–644)	Tian et al. (2022), Zhu et al. (2022)	Gamma
Platelet count decreased	3,588 (2,870–4,306)	Tian et al. (2022), Zhu et al. (2022)	Gamma
Laboratory	97 (78–116)	Zhu et al. (2022)	Gamma
Tumor imaging	208 (166–250)	Zhu et al. (2022)	Gamma
Administration	48 (38–58)	Zhu et al. (2022)	Gamma
Best supportive care	142 (114–170)	Zhu et al. (2022)	Gamma
Terminal care per patient	1,833 (1,466–2,200)	Zhu et al. (2022)	Gamma
Discount rate	0.05 (0–0.08)	Zhao et al. (2022)	Uniform

Abbreviation: OS, overall survival; PFS, progression-free survival; PD, disease progressed; AEs, adverse events; TC, tislelizumab plus chemotherapy.

### 2.4 Cost and utility

Direct medical expenses including the costs of drugs, laboratory testing, Tumor imaging, best supportive care (BST), terminal care, administration, and AE management were taken into consideration for this model (Zhu et al., 2022). Grade ≥3 AEs were included in the model when they occurred in more than 5% of patients, as they have a significant impact on survival and cost (Tian et al., 2022; Zhu et al., 2022). All costs were derived based on data from local hospitals or prior publications. In the established Markov model, health state was assigned a utility value ranging from 1 (perfect health) to 0 (deceased), with these values having been derived from the literature given that utility values were not reported in the RATIONALE-309 trial. For R/M NPC patients in this study, the PFS and PD states were assigned respective utility values of 0.65 and 0.52. A disutility value was also applied based on prior publications for grade ≥3 AEs (Table 1) (Zhu et al., 2022). All costs are exchanged into US dollars at the rate of $1 = ¥7.0848.

### 2.5 Sensitivity and subgroup analyses

Initially, one-way sensitivity analysis is conducted by individually varying each input parameter to 20% above or below baseline values as a means of assessing model robustness. This approach enabled the identification of the variables with the greatest impact on the economic outcomes derived from the established model. Then, probabilistic sensitivity analysis is conducted with a Monte Carlo simulation and 10,000 iterations in which all input parameters were simultaneously samples from across defined probability distributions. All costs were sampled based on a gamma distribution, while utility values and probabilities were samples based on beta distributions. The odds of tislelizumab plus chemotherapy being cost-effective relative to chemotherapy alone at a $36,289/QALY WTP threshold, the results of these 10,000 simulations were used to establish cost-effectiveness acceptability curves. Subgroup analysis were also conducted to assess the impact of different patient characteristics on model outcomes. Subgroup analysis is performed by modualting HRs for OS and PFS for different subgroups included in the RATIONALE-309 trial.

## 3 Results

### 3.1 Base-case analysis

Using the established Markov model, R/M NPC patients treated with tislelizumab plus chemotherapy achieved 4.86 LYs and 2.72 QALYs at a cost of $33,693, while patients treated with chemotherapy alone achieved 3.00 LYs and 1.67 QALYs at a cost of $15,982. Relative to chemotherapy alone, the combined tislelizumab plus chemotherapy regimen was associated with an incremental cost of $17,711 and an additional 1.86 LYs and 1.05 QALYs, for respective ICER values of $9,504/LY and $16,859/QALY (Table 2).

**TABLE 2:** Results of the base-case analysis.

Treatment	Total cost, $	Overall LYs	Overall QALYs	ICER, $		INHB, QALY
				per LY	per QALY	
Chemotherapy	15,982	3.00	1.67	Reference	Reference	Reference
Tislelizumab plus Chemotherapy	33,693	4.86	2.72	9,504	16,859	0.56

Abbreviation: LYs, life-years; QALYs, quality-adjusted life-years; ICER, incremental cost-effectiveness ratio; INHB, incremental net health benefits.

### 3.2 Sensitivity analysis

**FIGURE 1:**
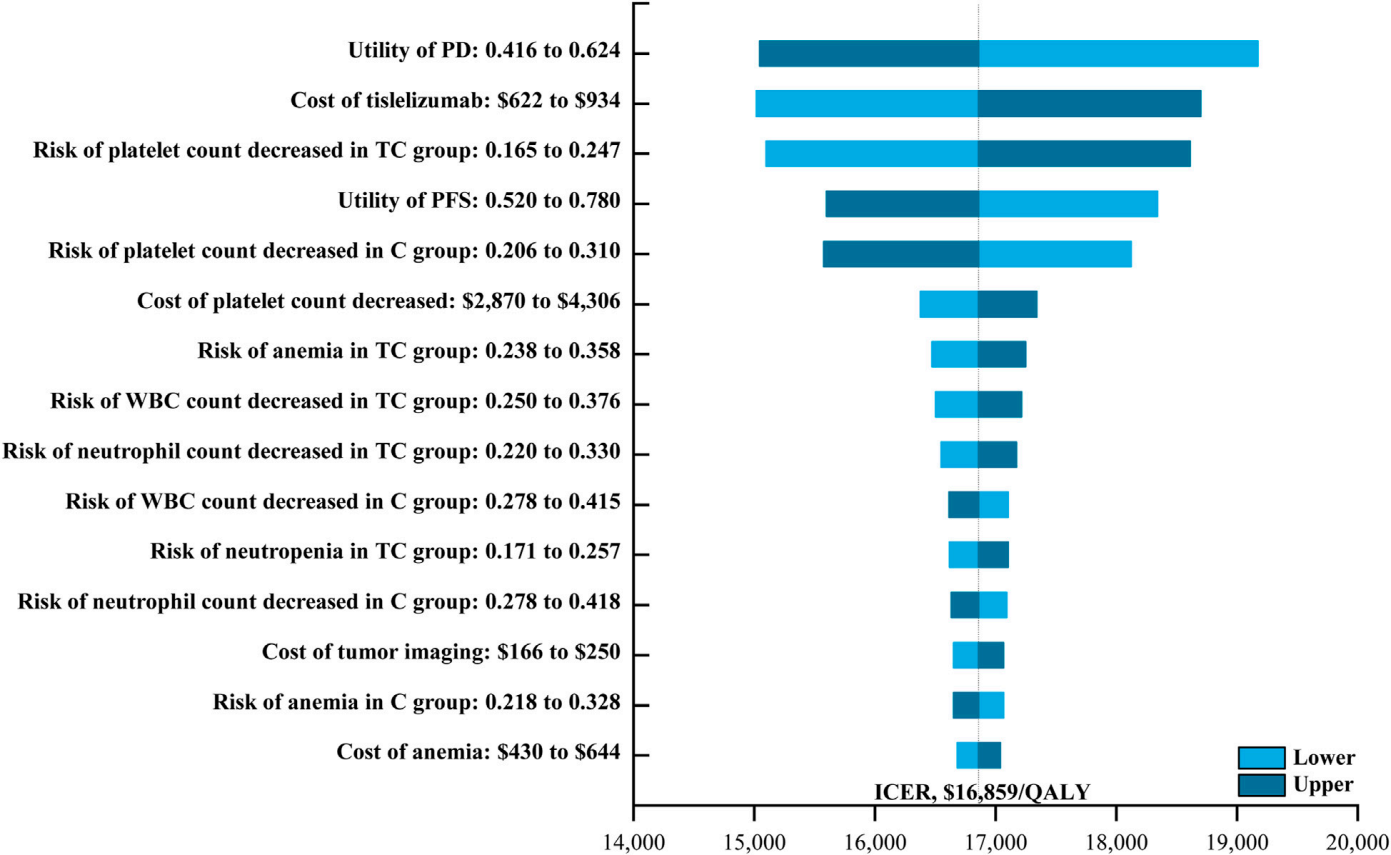
The One-way Sensitivity Analyses for Tislelizumab plus Chemotherapy Strategy Compared to Chemotherapy Alone Strategy. Abbreviation: PD, progressive disease; TC, tislelizumab plus chemotherapy; PFS, progression-free survival; C, chemotherapy; WBC, White blood cell; ICER, incremental cost-effectiveness ratio; QALY, quality-adjusted life-year.

**FIGURE 2:**
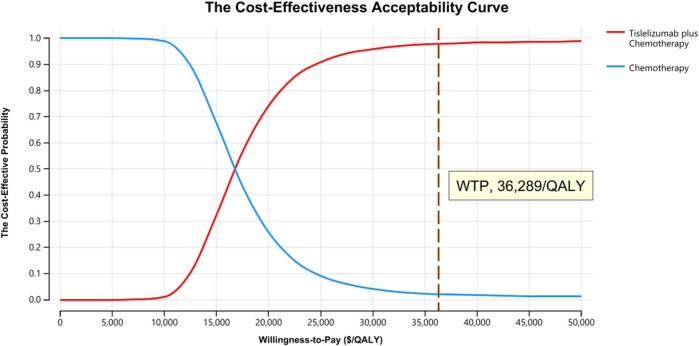
The Cost-effectiveness Acceptability Curves for Tislelizumab plus Chemotherapy Strategy Compared to Chemotherapy Alone Strategy. Abbreviation: QALY, quality-adjusted life-year.

One-way sensitivity analysis was next performed as a means of gauging the sensitivity of model outputs to changes in the selected inputs (Figure 1). This approach revealed that utility values, the cost of tislelizumab, and the incidence of platelet count decreased had the greatest impact on ICER values when comparing tislelizumab plus chemotherapy to chemotherapy alone. The ICER value for these one-way sensitivity analysis ranged from $15,045.18/QALY to $19,170.51/QALY. Model outcomes were largely unaffected by the disutility of AEs or the costs of chemotherapy or BSC. The cost-effectiveness acceptability curves revealed that the odds of tislelizumab plus chemotherapy group being cost-effective rose with increases in the WTP threshold (Figure 2). At a WTP threshold of $36,289/QALY, tislelizumab plus chemotherapy exhibited a 97.9% chance of being cost-effective (Supplementary Figure S3).

### 3.3 Subgroup analysis

All subgroups exhibited a superior reduction in progressive disease risk in response to tislelizumab plus chemotherapy treatment, with ICERs ranging from $9,698/QALY to $15,736/QALY when comparing this combination regimen to chemotherapy alone. Probabilistic sensitivity analysis demonstrated a clear trend toward tislelizumab plus chemotherapy yielding better cost-effectiveness outcomes with a 98.4%–99.3% chance of being cost-effective. Notably, this combination regimen was confirmed to be more cost-effective in former smokers, patients with Eastern Cooperative Oncology Group performance status of 1, individuals with primary metastatic disease, individuals without baseline liver metastasis, and patients with an EBV DNA level < 500 IU/mL (Table 3).

**TABLE 3:** Results of subgroup analyses.

Subgroup	PFS HR (95% CI)	ICER, $/QALY	INHB, QALYs	Cost-effectiveness probability of tislelizumab plus chemotherapy at WTP, %
				$36,289/QALY
Age, years				
< 65	0.45 (0.33–0.62)	13,790 (10,335–17,418)	0.64 (0.54–0.71)	98.6%
≥ 65	1.91 (0.73; 5.02)	NA	NA	NA
Sex				
Male	0.51 (0.36–0.71)	12,403 (8,971–16,375)	0.67 (0.58–0.74)	98.9%
Female	0.44 (0.23–0.83)	14,044 (7,476–21,919)	0.63 (0.43–0.78)	98.3%
ECOG PS				
0	0.46 (0.28–0.74)	13,543 (8,567–19,435)	0.65 (0.50–0.76)	98.7%
1	0.51 (0.35–0.75)	12,403 (8,437–16,710)	0.67 (0.57–0.76)	99.1%
Smoking status				
Never	0.38 (0.25–0.58)	15,736 (11,027–20,858)	0.59 (0.49–0.70)	98.4%
Current	0.41 (0.10–1.67)	NA	NA	NA
Former	0.66 (0.41–1.06)	9,698 (5,298–14,852)	0.73 (0.62–0.83)	99.3%
Diseased status				
Primary metastatic	0.53 (0.39–0.71)	11,986 (8,971–15,432)	0.67 (0.60–0.74)	99.0%
Recurrent	0.10 (0.01–0.86)	NA	NA	NA
Liver metastases at baseline				
Yes	0.48 (0.31–0.74)	13,069 (8,567–18,178)	0.65 (0.53–0.76)	98.9%
No	0.53 (0.35–0.80)	11,986 (7,821–16,710)	0.67 (0.57–0.78)	99.0%
EBV DNA level				
<500 IU/mL	0.55 (0.28–1.07)	11,589 (5,219–19,435)	0.69 (0.50–0.83)	99.0%
≥500 IU/mL	0.46 (0.32–0.64)	13,543 (10,010–17,791)	0.65 (0.54–0.72)	98.7%
PD-L1 expression at tumor cell				
<10%	0.46 (0.26–0.81)	13,543 (7,704–20,363)	0.65 (0.48–0.78)	98.7%
≥10%	0.47 (0.32–0.68)	13,301 (9,399–17,791)	0.65 (0.54–0.74)	98.8%

NA, the sample size was too small and was not further calculated.

Abbreviation: CI, confidence interval; PFS HR, progression-free survival hazard ratio; ICER, incremental cost-effectiveness ratio; INHB, incremental net health benefits; QALY, quality-adjusted life-year; WTP, Willingness-to-pay; ECOG PS, eastern cooperative oncology group performance status; EBV DNA, Epstein-Barr virus deoxyribonucleic acid; PD-L1, programmed cell death-Ligand 1.

## 4 Discussion

Immunotherapy have recently emerged as a key component of R/M NPC patient treatment regimens, contributing to improved patient survival at the expense of higher healthcare costs (Mai et al., 2021; Yang et al., 2021; Adkins and Haddad, 2022; Tan et al., 2022). Pharmacoeconomic studies provide an opportunity to evaluate specific drugs and treatment regimens based on their efficacy and economic utility, with cost-effectiveness analysis being the most commonly employed avenue of pharmacoeconomic investigation (Verma et al., 2018). The present study is the first to our knowledge to use the most recent clinical evidence to develop a model-based approach to assessing the cost-effectiveness of tislelizumab plus chemotherapy as a treatment for R/M NPC from the perspective of the Chinese healthcare system.

In the present study, tislelizumab plus chemotherapy yielded significant improvements in both LYs and QALYs relative to chemotherapy alone (1.86 LYs and 1.05 QALYs, respectively) at an incremental cost of $17,711, for corresponding ICER values of $9,504/LY and $16,859/QALY. One-way sensitivity analysis revealed that the utility of PD, the cost of tislelizumab, and the risk of platelet count decreased in patients undergoing tislelizumab plus chemotherapy treatment were the three factors with the greatest impact on the cost-effectiveness of this combination regimen. However, tislelizumab plus chemotherapy remained cost-effective even when these three factors were varied by 20% relative to baseline levels. Probabilistic sensitivity analysis also suggested that tislelizumab plus chemotherapy had a 97.9% chance of being cost-effective at WTP threshold of $36,289/QALY. In China, medical insurance policies were also worthy of attention. At present, tislelizumab is not covered by medical insurance when it is used in patients with recurrent or metastatic nasopharyngeal carcinoma in China. When we considered the local health insurance policy, the co-payment rate for gemcitabine, cisplatin, and capecitabine is 20%, 0%, and 5%, respectively. With an ICER of $16,140.79/QALY, tislelizumab plus chemotherapy remained cost-effective. In prior studies, both camrelizumab and toripalimab, which were developed by Chinese companies, were found to be cost-effective alternatives to chemotherapy when used to treat R/M NPC combining with chemotherapy (Zhu et al., 2022). Camrelizumab plus chemotherapy and toripalimab plus chemotherapy yielded respective ICERs of $20,438/QALY and $19,726/QALY, both of which were well below the Chinese WTP threshold (Zhu et al., 2022). This consistency in findings across different immunotherapeutic regimens further supports the value of tislelizumab as a cost-effective component of treatment regimens for patients suffering from R/M NPC.

However, with the promotion of precision cancer treatment, it is much more in line with the current treatment strategy to screen the appropriate population and use the suitable drugs to achieve individualized treatment. EBV infections are extremely common in patients with non-keratinizing NPC, serving as a driver of this form of cancer (Tsao et al., 2017; Adkins and Haddad, 2022). Non-keratinizing cases account for 75%–95% of NPC cases in different regions (Lee et al., 2019). EBV screening in the endemic population can improve the staging distribution of NPC and reduce cancer-related mortality. The RATIONALE-309 trial noted a significant PFS benefit in patients exhibiting plasma EBV DNA positive (≥500 IU/mL) group [HR: 0.46 (95% CI: 0.32, 0.64)], whereas no corresponding benefit was noted in plasma EBV DNA negative (<500 IU/mL) group [HR: 0.55 (95% CI: 0.28, 1.07)] (Yang et al., 2023). While PD-L1 expression levels do not precisely correspond to tumor therapeutic responses, the levels of this key immune checkpoint-related protein are nonetheless invaluable as a predictive biomarker associated with patient outcomes following immunotherapy (Jiang et al., 2019; Doroshow et al., 2021). Differences in drug efficacy have been noted as a function of PD-L1 status, but these benefits are not consistent across trials and cancer types. The KEYNOTE-826 trial, for example, found that a combination of pembrolizumab plus chemotherapy was beneficial to the survival of PD-L1-positive patients with persistent, recurrent, or metastatic cervical cancer irrespective of bevacizumab treatment status, whereas PD-L1 negative patients did not exhibit any comparable PFS (HR, 0.94; 95% CI; 0.52 to 1.70; *p* < 0.05) or OS (HR, 1.00; 95% CI; 0.53 to 1.89; *p* < 0.05) benefits (Colombo et al., 2021). Nevertheless, cemiplimab can significant improved PFS compared chemotherapy in PD-L1 negative subgroup (HR, 0.64; 95% CI; 0.49 to 0.84; *p* < 0.05) (Tewari et al., 2022).

The results from the RATIONALE-309 trial were also used to conduct subgroup analysis, highlighting a strength of the present analysis over prior studies in which treatments were not evaluated for particular subsets of patients (Tian et al., 2022; Zhu et al., 2022). ICER values were below the WTP threshold for most subgroups, in line with the results of the base case analysis, emphasizing that tislelizumab plus chemotherapy represents a cost-effective treatment option irrespective of EBV DNA level, PD-L1 expression levels in tumor cells, and liver metastases at baseline in patients. The probability that tislelizumab plus chemotherapy will be cost-effective in each of these subgroups is 99.0%, 98.7%, 98.7%, 98.8%, 98.9%, and 99.0%, respectively. These results are attributable to the high efficacy and low cost of tislelizumab plus chemotherapy.

There are several strengths of this study that deserve to be highlighted. First, this study is the first cost-effectiveness analysis comparing tislelizumab with chemotherapy and chemotherapy alone. Second, the clinical and economic outcomes discussed herein were simulated with Markov models. Based on the reconstruction of individual patient data, the calculations of transition probabilities, and the model assumptions discussed herein, other researchers in the future will be able to readily reconstruct this model to validate or build upon these study conclusions. Third, this cost-effectiveness analysis was conducted for a range of patient subgroups, thereby supporting individualized treatment planning. Lastly, as the cost-effectiveness of tislelizumab plus chemotherapy was evaluated from a Chinese healthcare system perspective, these results may help inform the decision-making of clinicians, government workers, and healthcare institutions in China while also facilitating the update of the CSCO guidelines.

This analysis is subject to several limitations. Firstly, models were constructed based upon the short-term follow-up data from the RATIONALE-309 trial such that long-term survival data were inferred, potentially impacting the accuracy of these results. At present, however, there is no way to avoid this methodological error, and efforts to improve study accuracy thus hinge on improvements to model precision. Secondly, the RATIONALE-309 trial did not include quality of life-related data such that the utility values used herein were based on prior publications and may differ from the actual values. In addition, with regard to the cost, we only considered AEs of grade 3 or higher with an incidence greater than 5%, which may underestimate the cost of managing AEs.

## 5 Conclusion

In summary, a Markov model was herein used to explore the cost-effectiveness of tislelizumab plus chemotherapy when treating R/M NPC. At the $36,289/QALY WTP threshold and under current drug pricing, these results suggest that this therapeutic combination is a cost-effective option for R/M NPC patient treatment in China. These data provide a rational basis that can be used by clinicians and patients to select appropriate drug treatment strategies, while also informing medical reimbursement policy development.

## Data Availability

The original contributions presented in the study are included in the article/Supplementary Material, further inquiries can be directed to the corresponding author.

## References

[B1] Adkins D. R. Haddad R. I. (2022). Clinical trial data of Anti-PD-1/PD-L1 therapy for recurrent or metastatic nasopharyngeal Carcinoma: a review. Cancer Treat. Rev. 109, 102428. 10.1016/j.ctrv.2022.102428 35753157

[B2] Chen Y. P. Chan A. T. C. Le Q. T. Blanchard P. Sun Y. Ma J. (2019). Nasopharyngeal carcinoma. Lancet 394 (10192), 64–80. 10.1016/S0140-6736(19)30956-0 31178151

[B3] Colombo N. Dubot C. Lorusso D. Caceres M. V. Hasegawa K. Shapira-Frommer R. (2021). Pembrolizumab for persistent, recurrent, or metastatic cervical cancer. N. Engl. J. Med. 385 (20), 1856–1867. 10.1056/NEJMoa2112435 34534429

[B4] CSCO (2023) Available at: http://www.csco.org.cn/cn/index.aspx.

[B5] Doroshow D. B. Bhalla S. Beasley M. B. Sholl L. M. Kerr K. M. Gnjatic S. (2021). PD-L1 as a biomarker of response to immune-checkpoint inhibitors. Nat. Rev. Clin. Oncol. 18 (6), 345–362. 10.1038/s41571-021-00473-5 33580222

[B6] Hoyle M. W. Henley W. (2011). Improved curve fits to summary survival data: application to economic evaluation of health technologies. BMC Med. Res. Methodol. 11, 139. 10.1186/1471-2288-11-139 21985358 PMC3198983

[B7] Husereau D. Drummond M. Augustovski F. de Bekker-Grob E. Briggs A. H. Carswell C. (2022). Consolidated health economic evaluation reporting Standards 2022 (CHEERS 2022) statement: updated reporting guidance for health economic evaluations. Value Health 25 (1), 3–9. 10.1016/j.jval.2021.11.1351 35031096

[B8] Jiang Y. Chen M. Nie H. Yuan Y. (2019). PD-1 and PD-L1 in cancer immunotherapy: clinical implications and future considerations. Hum. Vaccin Immunother. 15 (5), 1111–1122. 10.1080/21645515.2019.1571892 30888929 PMC6605868

[B9] Jin Y. Shi Y. X. Cai X. Y. Xia X. Y. Cai Y. C. Cao Y. (2012). Comparison of five cisplatin-based regimens frequently used as the first-line protocols in metastatic nasopharyngeal carcinoma. J. Cancer Res. Clin. Oncol. 138 (10), 1717–1725. 10.1007/s00432-012-1219-x 22684794 PMC11824688

[B10] Lee A. W. M. Ng W. T. Chan J. Y. W. Corry J. Mäkitie A. Mendenhall W. M. (2019). Management of locally recurrent nasopharyngeal carcinoma. Cancer Treat. Rev. 79, 101890. 10.1016/j.ctrv.2019.101890 31470314

[B11] Liu K. Zhu Y. Zhu H. (2022). Immunotherapy or targeted therapy as the first-line strategies for unresectable hepatocellular carcinoma: a network meta-analysis and cost-effectiveness analysis. Front. Immunol. 13, 1103055. 10.3389/fimmu.2022.1103055 36713376 PMC9874298

[B12] Mai H. Q. Chen Q. Y. Chen D. Hu C. Yang K. Wen J. (2021). Toripalimab or placebo plus chemotherapy as first-line treatment in advanced nasopharyngeal carcinoma: a multicenter randomized phase 3 trial. Nat. Med. 27 (9), 1536–1543. 10.1038/s41591-021-01444-0 34341578

[B13] NMPA (2023) Available at: https://www.nmpa.gov.cn.

[B14] Sung H. Ferlay J. Siegel R. L. Laversanne M. Soerjomataram I. Jemal A. (2021). Global cancer statistics 2020: GLOBOCAN estimates of incidence and mortality worldwide for 36 cancers in 185 countries. CA Cancer J. Clin. 71 (3), 209–249. 10.3322/caac.21660 33538338

[B15] Tan L. L. Y. Le Q. T. Lee N. Y. Y. Chua M. L. K. (2022). JUPITER-02 trial: advancing survival for recurrent metastatic nasopharyngeal carcinoma and next steps. Cancer Commun. (Lond) 42 (1), 56–59. 10.1002/cac2.12248 34918497 PMC8753315

[B16] Tewari K. S. Monk B. J. Vergote I. Miller A. de Melo A. C. Kim H. S. (2022). Survival with cemiplimab in recurrent cervical cancer. N. Engl. J. Med. 386 (6), 544–555. 10.1056/NEJMoa2112187 35139273

[B17] Tian K. Han J. Wang Z. Chen J. (2022). Immune checkpoint inhibition in first-line treatment for recurrent or metastatic nasopharyngeal carcinoma: a CAPTAIN-1st and JUPITER-02 trial-based cost-effectiveness analysis. Oral Oncol. 128, 105842. 10.1016/j.oraloncology.2022.105842 35428025

[B18] Tsao S. W. Tsang C. M. Lo K. W. (2017). Epstein-Barr virus infection and nasopharyngeal carcinoma. Philos. Trans. R. Soc. Lond B Biol. Sci. 372 (1732), 20160270. 10.1098/rstb.2016.0270 28893937 PMC5597737

[B19] Verma V. Sprave T. Haque W. Simone C. B. Chang J. Y. Welsh J. W. (2018). A systematic review of the cost and cost-effectiveness studies of immune checkpoint inhibitors. J. Immunother. Cancer 6 (1), 128. 10.1186/s40425-018-0442-7 30470252 PMC6251215

[B20] Wang T. L. Tan Y. O. (1991). Cisplatin and 5-fluorouracil continuous infusion for metastatic nasopharyngeal carcinoma. Ann. Acad. Med. Singap 20 (5), 601–603. 1781642

[B21] Wong K. C. W. Hui E. P. Lo K. W. Lam W. K. J. Johnson D. Li L. (2021). Nasopharyngeal carcinoma: an evolving paradigm. Nat. Rev. Clin. Oncol. 18 (11), 679–695. 10.1038/s41571-021-00524-x 34194007

[B22] Yang Y. Pan J. Wang H. Zhao Y. Qu S. Chen N. (2023). Tislelizumab plus chemotherapy as first-line treatment for recurrent or metastatic nasopharyngeal cancer: a multicenter phase 3 trial (RATIONALE-309). Cancer Cell 41 (6), 1061–1072.e4. 10.1016/j.ccell.2023.04.014 37207654

[B23] Yang Y. Qu S. Li J. Hu C. Xu M. Li W. (2021). Camrelizumab versus placebo in combination with gemcitabine and cisplatin as first-line treatment for recurrent or metastatic nasopharyngeal carcinoma (CAPTAIN-1st): a multicentre, randomised, double-blind, phase 3 trial. Lancet Oncol. 22 (8), 1162–1174. 10.1016/S1470-2045(21)00302-8 34174189

[B24] Zhang L. Huang Y. Hong S. Yang Y. Yu G. Jia J. (2016). Gemcitabine plus cisplatin versus fluorouracil plus cisplatin in recurrent or metastatic nasopharyngeal carcinoma: a multicentre, randomised, open-label, phase 3 trial. Lancet 388 (10054), 1883–1892. 10.1016/S0140-6736(16)31388-5 27567279

[B25] Zhao M. Pan X. Yin Y. Hu H. Wei J. Bai Z. (2022). Cost-effectiveness analysis of five systemic treatments for unresectable hepatocellular carcinoma in China: an economic evaluation based on network meta-analysis. Front. Public Health 10, 869960. 10.3389/fpubh.2022.869960 35493395 PMC9051228

[B26] Zhu Y. Liu K. Ding D. Wang K. Liu X. Tan X. (2022). Chemo-immunotherapy regimes for recurrent or metastatic nasopharyngeal carcinoma: a network meta-analysis and cost-effectiveness analysis. Front. Pharmacol. 13, 858207. 10.3389/fphar.2022.858207 35668931 PMC9163401

