# Supplementary material for: Cost-effectiveness analysis of first-line tislelizumab plus chemotherapy for recurrent or metastatic nasopharyngeal cancer
Source: Front Pharmacol. 2023 Oct 30;14:1265784. doi: 10.3389/fphar.2023.1265784 (PMC10642263; doi:10.3389/fphar.2023.1265784)
Supplement: Supplementary file 1 [file DataSheet1.PDF]

Figure S1. Model Structure.

Figure S2. Kaplan-Meier Curve Fitting and Extrapolation.

Figure S3. Probability Sensitivity Analysis Scatter Plot.

Table S1. The CHEERS 2022 checklist.

Table S2. Details of Treatment Strategy and Unit Costs.

Table S3. Summary of Statistical Goodness-of-fit of K-M Curve.

Figure S1. Model Structure.

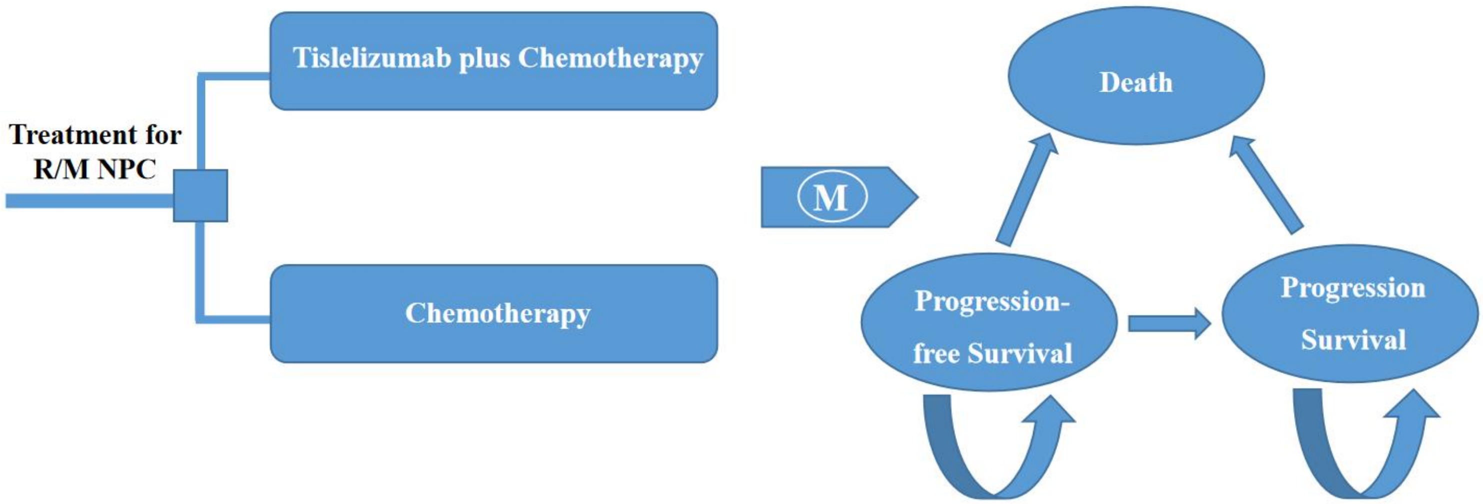

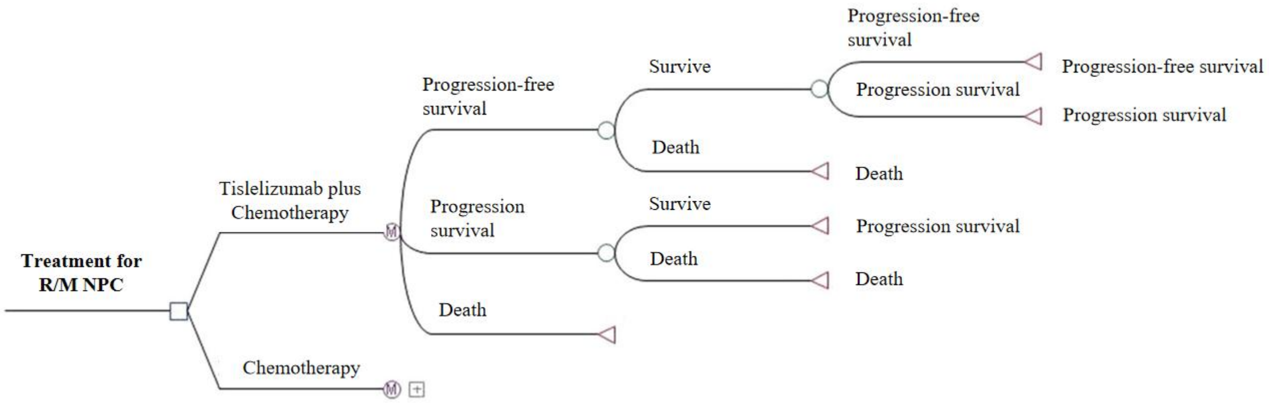

Abbreviation: R/M NPC, recurrent or metastatic nasopharyngeal carcinoma; M, Markov.

Figure S2. Kaplan-Meier Curve Fitting and Extrapolation.

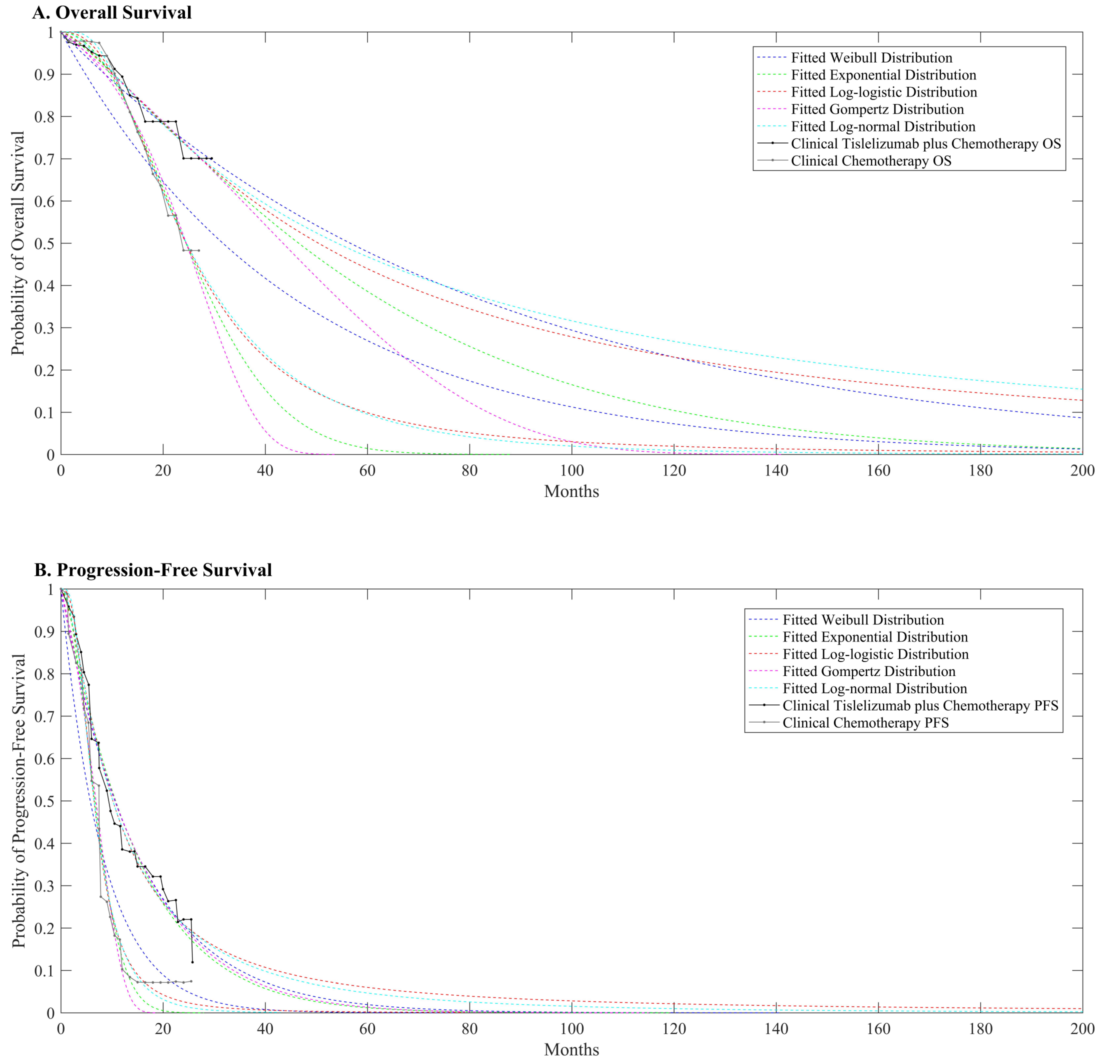

Abbreviation: OS, overall survival; PFS, progression-free survival.

Kaplan-meier curve fitting and extrapolation for overall survival (A) and progression-free survival (B), respectively.

Figure S3. Probability Sensitivity Analysis Scatter Plot.

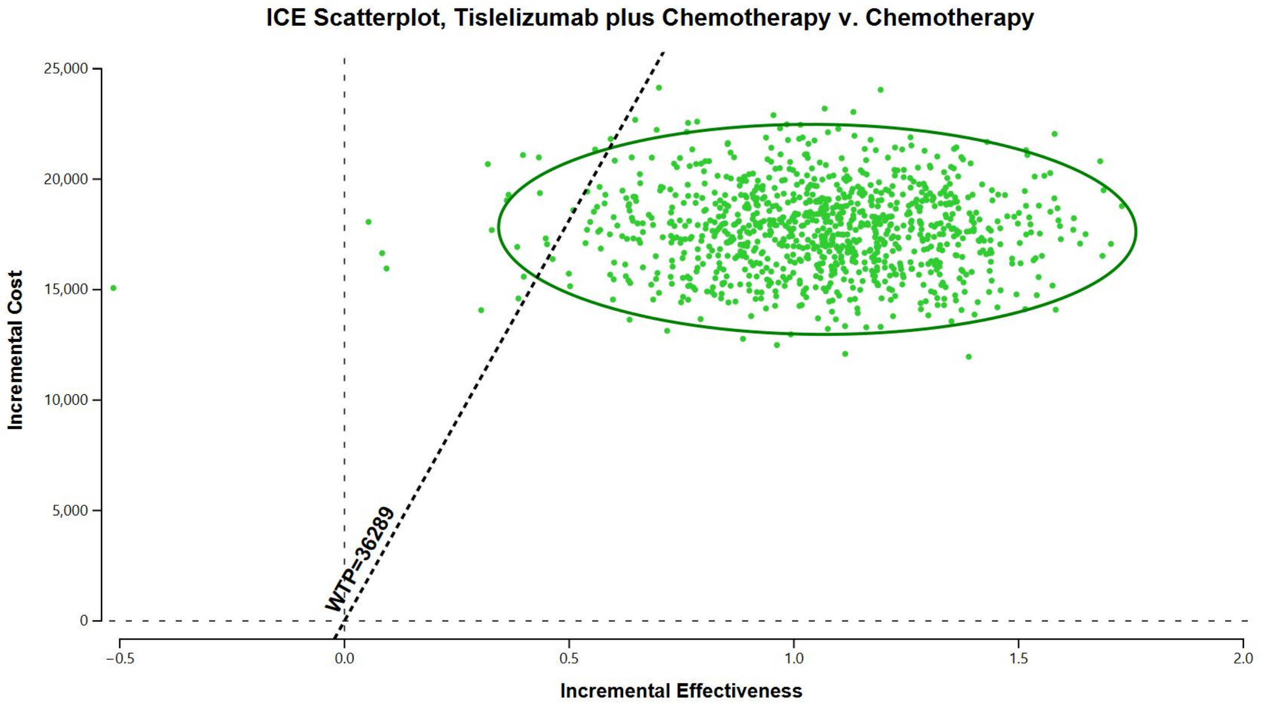

Abbreviation: WTP, willingness-to-pay.

Each point in the diagram represents a simulation result of 10,000 Monte Carlo simulations. The ellipse represents the 95% CI and the dotted line represents WTP of $36,289/QALY. Below the dotted line (green dot) is the probability that tislelizumab plus chemotherapy will be cost-effective.

Table S1. The CHEERS 2022 checklist.

Section/item	Item No	Recommendation	Reported?
**Title and abstract**			
Title	1	Identify the study as an economic evaluation and specify the interventions being compared.	Yes
Abstract	2	Provide a structured summary that highlights context, key methods, results, and alternative analyses.	Yes
**Introduction**			
Background and objectives	3	Give the context for the study, the study question, and its practical relevance for decision making in policy or practice.	Yes
**Methods**			
Health economic analysis plan	4	Indicate whether a health economic analysis plan was developed and where available.	Yes
Study population	5	Describe characteristics of the study population (such as age range, demographics, socioeconomic, or clinical characteristics).	Yes
Setting and location	6	Provide relevant contextual information that may influence findings.	Yes
Comparators	7	Describe the interventions or strategies being compared and why chosen.	Yes
Perspective	8	State the perspective(s) adopted by the study and why chosen.	Yes
Time horizon	9	State the time horizon for the study and why appropriate.	Yes

Discount rate	10	Report the discount rate(s) and reason chosen.	Yes
Selection of outcomes	11	Describe what outcomes were used as the measure(s) of benefit(s) and harm(s)	Yes
Measurement of outcomes	12	Describe how outcomes used to capture benefit(s) and harm(s) were measured.	Yes
Valuation of outcomes	13	Describe the population and methods used to measure and value outcomes.	Yes
Measurement and valuation of resources and costs	14	Describe how costs were valued.	Yes
Currency, price date, and conversion	15	Report the dates of the estimated resource quantities and unit costs, plus the currency and year of conversion.	Yes
Rationale and description of model	16	If modelling is used, describe in detail and why used. Report if the model is publicly available and where it can be accessed.	Yes
Analytics and assumptions	17	Describe any methods for analysing or statistically transforming data, any extrapolation methods, and approaches for validating any model used.	Yes
Characterizing heterogeneity	18	Describe any methods used for estimating how the results of the study vary for subgroups.	Yes
Characterizing distributional effects	19	Describe how impacts are distributed across different individuals or adjustments made to reflect priority populations.	Yes
Characterizing uncertainty	20	Describe methods to characterise any sources of uncertainty in the analysis.	Yes
Approach to engagement with patients and others affected by the study	21	Describe any approaches to engage patients or service recipients, the general public, communities, or stakeholders (such as clinicians or payers) in the design of the study.	Not applicable
**Results**			

Study parameters	22	Report all analytic inputs (such as values, ranges, references) including uncertainty or distributional assumptions.	Yes
Summary of main results	23	Report the mean values for the main categories of costs and outcomes of interest and summarise them in the most appropriate overall measure.	Yes
Effect of uncertainty	24	Describe how uncertainty about analytic judgments, inputs, or projections affect findings. Report the effect of choice of discount rate and time horizon, if applicable.	Yes
Effect of engagement with patients and others affected by the study	25	Report on any difference patient/service recipient, general public, community, or stakeholder involvement made to the approach or findings of the study.	Not applicable
**Discussion**			
Study findings, limitations, generalizability, and current knowledge	26	Report key findings, limitations, ethical or equity considerations not captured, and how these could affect patients, policy, or practice.	Yes
**Other**			
Source of funding	27	Describe how the study was funded and any role of the funder in the identification, design, conduct, and reporting of the analysis	Yes
Conflicts of interest	28	Report authors conflicts of interest according to journal or International Committee of Medical Journal Editors requirements.	Yes

Reference:
Husereau D, Drummond M, Augustovski F, de Bekker-Grob E, Briggs AH, Carswell C, et al. Consolidated Health Economic Evaluation Reporting Standards 2022 (CHEERS 2022) Statement: Updated Reporting Guidance for Health Economic Evaluations. Value Health. 2022;25(1):3-9.

Table S2. Details of Treatment Strategy and Unit Costs.

Drug	Dose	Time	Unit costs, $
Tislelizumab plus Chemotherapy	Tislelizumab , 200mg	Administered tislelizumab 200 mg intravenously every 3 weeks	1.9443
	Gemcitabine, 1000 mg/m^2	Administered gemcitabine 1000 mg/m^2 intravenously was given on Day 1 and Day 8, and cisplatin 80 mg/m2 on Day 1 every 3 weeks for 6 cycles	0.0056
	Cisplatin, 80 mg/m^2		0.1125
Chemotherapy	Gemcitabine, 1000 mg/m^2	Administered gemcitabine 1000 mg/m^2 intravenously was given on Day 1 and Day 8, and cisplatin 80 mg/m2 on Day 1 every 3 weeks for 6 cycles	0.0056
	Cisplatin, 80 mg/m^2		0.1125
Capecitabine	Capecitabine, 1250 mg/m^2	Administered oral 1250 mg bid d1-14 3weeks	0.0006

Table S3. Summary of Statistical Goodness-of-fit of K-M Curve.

	Exponential	Weibull	Gompertz	Log-logistic	Log-normal
**Tislelizumab plus chemotherapy OS curve**					
AIC	11.5172	**9.7006**	11.5701	11.5006	11.4546
BIC	13.6062	**10.7452**	13.6591	13.5896	13.5436
**Chemotherapy OS curve**					
AIC	14.9213	**13.2085**	15.0899	14.8613	14.7729
BIC	16.8102	**14.1529**	16.9788	16.7501	16.6618
**Tislelizumab plus chemotherapy PFS curve**					
AIC	51.0579	**48.4012**	50.6389	50.5642	50.4566
BIC	53.7223	**49.7334**	53.3033	53.2286	53.1210
**Chemotherapy PFS curve**					
AIC	122.1687	**68.8646**	300.7074	83.5030	88.5164
BIC	124.6064	**70.0835**	303.1452	85.9408	90.9541

Abbreviation: OS, overall survival; PFS, progression-free survival; AIC, Akaike's information criterion; BIC, Bayesian information criterion.

The AIC and BIC value of the distributions and the visual fitting of the curves (Figure S2) showed that the Weibull distribution is probably the most reasonable parametric survival model. Weibull distribution is flexible and widely used for matching patients with three states over time because it can monotonously increase or decrease risk functions, and it is suitable for estimating events occurring during early follow-up work.
